# Transit time flow measurement guiding the surgical treatment for anomalous origin of the right coronary artery: A case report

**DOI:** 10.3389/fcvm.2022.975014

**Published:** 2022-10-20

**Authors:** Federica Jiritano, Angelo Leone, Francesco Greco, Mario Leporace, Carlo Bova, Vincenzo Aiello, Giuseppe Filiberto Serraino, Pasquale Mastroroberto

**Affiliations:** ^1^Cardiac Surgery Unit, Department of Experimental and Clinical Medicine, University “Magna Græcia” of Catanzaro, Catanzaro, Italy; ^2^Cath Lab Unit, Azienda Ospedaliera Santissima Annunziata Hospital, Cosenza, Italy; ^3^Radiology Unit, Azienda Ospedaliera Santissima Annunziata Hospital, Cosenza, Italy; ^4^Medical Unit, Azienda Ospedaliera Santissima Annunziata Hospital, Cosenza, Italy; ^5^Department of Clinical Medicine and Surgery, University of Naples Federico II, Naples, Italy

**Keywords:** coronary artery, anomalous coronary artery anatomy, ACAOS, transit time flowmetry (TTFM), case report

## Abstract

Anomalous origin of a coronary artery from the opposite sinus of Valsalva (ACAOS) in symptomatic patients is a rare but serious finding whose treatment consists of a surgical correction. The surgical treatment has a level of complexity that could vary from unroofing and ostioplasty to coronary artery bypass grafting. We present our management of a 59-year-old woman presenting with chest pain and dyspnea for right ACAOS with an interarterial route. The right coronary artery (RCA) was bypassed with the right internal thoracic artery. An intraoperative transit time flowmetry (TTFM) showed a competitive flow from the native RCA. RCA proximal ligation site was identified intraoperatively, considering the best mean graft flow (MGF) and the absence of ischemic events. The patient was discharged after a week without adverse events. The 1-year follow-up was uneventful. The intraoperative use of TTFM could guide the surgeon’s hand making straightforward the surgical treatment for ACAOS.

## Background

Anomalous origin of the coronary artery from the opposite sinus of Valsalva (ACAOS) with an interarterial course could lead to fatal outcomes (1). The recommended guideline for symptomatic patients with ACAOS is a surgical approach (2, 3). Several surgical procedures of different technical complexity have been proposed to treat ACAOS (1). New intraoperative technologies could set in in order to help the cardiac surgeon simplifying the operation. Herein, we report a case in which the use of transit time flow measurement (TTFM) simplified the surgical operation of a young woman with symptomatic right ACAOS and intramural and interarterial course (R-ACAOS-IM).

## Case presentation

**FIGURE 1:**
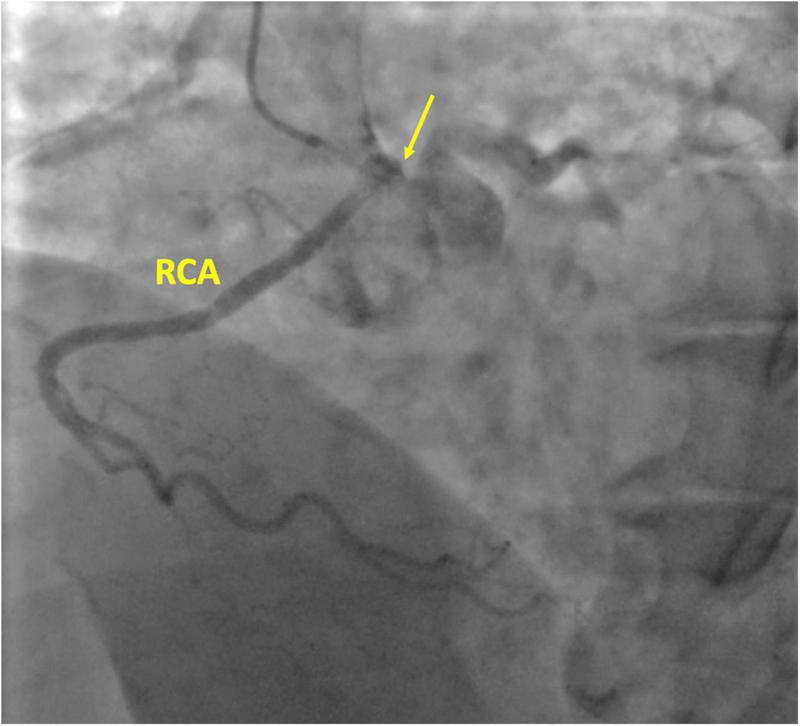
Coronary angiography of the right coronary artery (RCA). The yellow arrow is pointing the right coronary ostium originating from the left coronary sinus.

**FIGURE 2:**
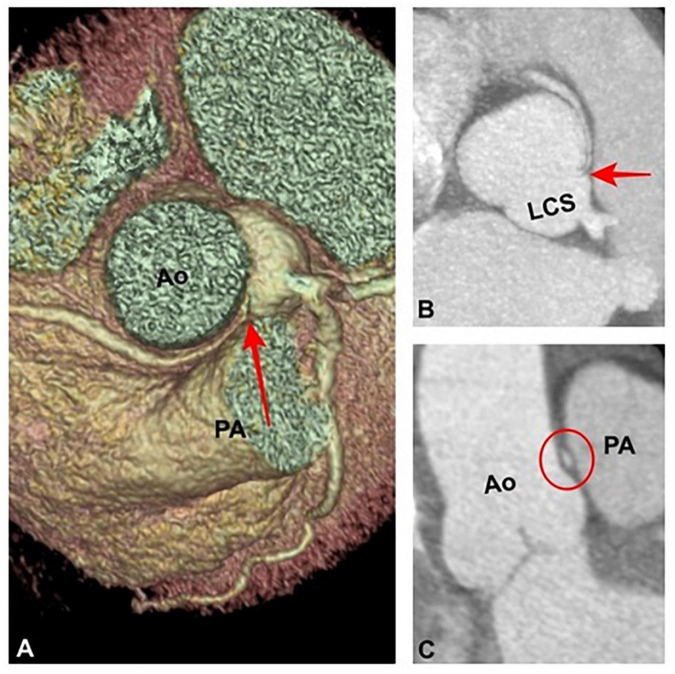
**(A)** Volume rendering and **(B)** maximum intensity projection reformations demonstrating an anomalous origin of the right coronary artery from the left coronary sinus and an its intramural and interarterial course (red arrows). **(C)** Coronal oblique image shows an “elliptic shape” (red circle) of the interarterial course of the right coronary artery (high-risk feature). Ao, Aorta. PA, Pulmonary Artery.

**FIGURE 3:**
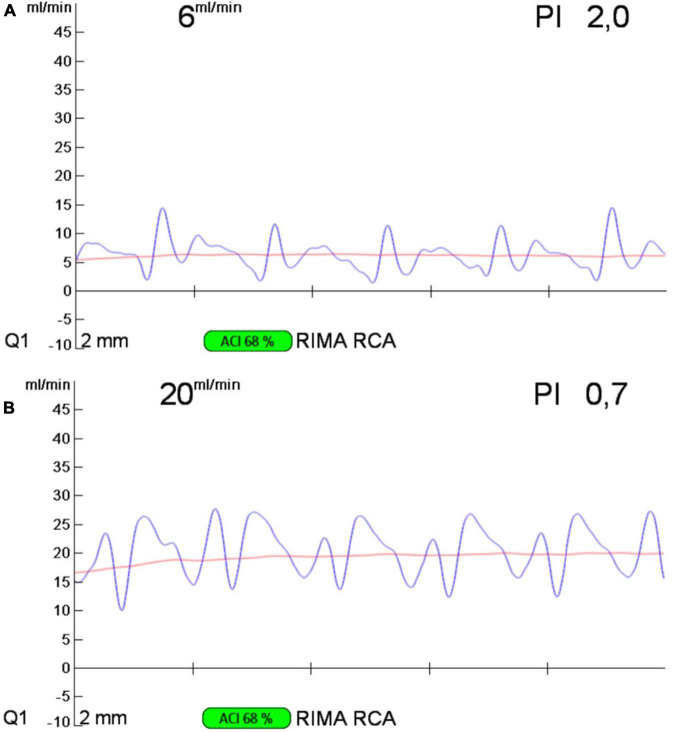
Transit time flow measurement of the RIMA-RCA bypass graft before **(A)** and after **(B)** proximal right coronary artery ligation. RIMA, right internal mammary artery; RCA, right coronary artery.

A 59-year-old white woman presented with chest pain and dyspnoea while sitting and watching a dramatic movie on TV. She had no preceding symptoms, and her past medical history was unremarkable. After admission to the emergency department, the symptoms resolved spontaneously. The myocardial necrosis markers (Troponin I and CK-MB) were not suggestive of myocardial infarction. The ECG revealed a normal sinus rhythm with no ischemic changes. She was transferred to the ward. Physical examination showed normal vital signs, regular heart rate and rhythm, and no extra sounds or murmurs. Transthoracic echocardiogram (TTE) showed preserved left ventricle ejection fraction (LVEF), normal wall motion, and no valvular diseases. The patient had a new onset chest pain while at rest. ECG showed new onset ST depression and T wave inversion in II, III, aVF, and V1-V3. She was quickly treated with sublingual nitroglycerine, and symptoms and ECG changes resolved. Repeat serial troponins resulted negative. She was scheduled for urgent cardiac catheterization with the diagnostic suspect of Prinzmetal angina. Coronary angiography showed unobstructed coronary arteries. After an acetylcholine test, coronary spasms were ruled out. Surprisingly, an R-ACAOS was also found (Figure 1). A CT coronary angiogram confirmed the origin of the right coronary artery (RCA) from the left sinus of Valsalva, with an intramural course and a malignant path between the aorta and the pulmonary artery (Figure 2). Despite being frightened, the patient consented to surgical correction of the coronary anomaly, which was performed by coronary artery bypass grafting. After a full sternotomy approach, the right internal mammary artery (RIMA) was harvested. Before cutting it distally, the TTFM was used to measure the RIMA flow, which was 22 mL/min. After dissecting the vessel distally, we noticed a nice flow with no sign of RIMA spasm. The RCA was isolated at its mid tract, and an off-pump coronary artery bypass grafting (OPCAB) has been performed with RIMA using a coronary shunt of 1.75 mm. After completing the anastomosis, transit time flowmetry (TTFM) showed a poor graft flow (6 mL/min with a pulsatility index (PI) of 2.0), indicating a competitive flow with the native RCA (Figure 3). Therefore, the RCA was proximally isolated and temporary occluded for 10 min in order to evaluate the best graft flow without ischemic drawbacks. At a graft flow of 20 mL/min with a PI of 0.7, the RCA was proximally ligated (Figure 3). The patient recovered uneventfully and was discharged 5 days after the operation. The patient was relieved that everything went nicely. One year after surgery, the follow-up evaluation of the patient confirmed the continuous absence of chest pain, dyspnoea, and a negative functional test with exercise stress echocardiography.

## Discussion

To our knowledge, this is the first report to highlight the intraoperative use of TTFM guiding the surgical correction of malignant R-ACAOS-IM.

Despite being founded just on retrospective single-center case series and registry data, Nord American and European guidelines recommend surgery for symptomatic patients (2, 3). However, several surgical procedures are available whose complexity ranges greatly (1, 4).

Unroofing is the procedure of choice for young patients (1). The operation consists of relocating and enlarging the functional orifice to the appropriate sinus and resecting the inner wall of the intramural segment (1). Possible drawbacks of the unroofing procedure are: (1) a post-procedure aortic insufficiency due to the damage or distortion of the intercoronary commissure; (2) possible aortic dissection with coronary occlusion due to the exposure of the layers of the aortic wall to the arterial pressure; (3) aortic or coronary incision after aggressive unroofing (1).

The pulmonary translocation has the goal of decompressing the interarterial course of the anomalous coronary artery by moving the pulmonary artery confluence away from the anomalous artery either laterally or anteriorly (1). It has a remarkable difficulty level.

Ostial reimplantation or reconstruction could be performed when there is little or no intramural component. It is technically challenging because it requires full mobilization of the coronary artery, avoiding its kinking (1).

Alternatively, a new ostium could be created through an incision in the proper aortic sinus and a pericardial patch. It requires a meticulous reconstruction of the coronary artery itself (1). This is probably the most technically challenging of all of the surgical options.

Acute coronary occlusion, scarring stenosis, or pseudo-aneurysms are early and late reported complications of ostioplasty and ostial reconstruction (1).

An indirect and straightforward way to treat R-ACAOS-IM is coronary bypass grafting. This strategy prevents the risks of the unroofing procedure, and the manipulation of the aorta, and could be performed off-pump, avoiding cardiopulmonary bypass. However, the flow from the anomalous RCA could become competitive, as the flow is unobstructed at rest (4). It could impair the patency of the graft and cause graft failure (4). However, some authors considered the RIMA-RCA graft inadequate to provide a sufficient blood supply and to cover the whole RCA myocardial territory (4). Usually, it requires a proximal ligation of the coronary artery to be successful, sacrificing the blood supply of the very proximal vessels originating from the RCA. In the present case, the TTFM proved to be an easy, safe and quick tool to evaluate the feasibility of proximal RCA ligation. As mentioned in the 2018 ESC/EACTS Guidelines on Myocardial Revascularization, through intraoperative assessment of coronary graft function, TTFM enables quality control in coronary artery bypass grafting (5). Two ultrasonic transducers fixed to a single flow probe are used in transit time ultrasound technology. One sensor sends out an ultrasonic signal that will travel through a pipe packed with fluid. The signal will be reflected by the opposite fixed reflector, which will be picked up by a second sensor. The flow velocity in the pipe will affect the transit time of the signal, which is the difference between the upstream and downstream transit times of the ultrasound beam. The blood flow volume is inversely proportional to the transit time difference. The quality of the target vessel, the distal run-off of the bypass, and the graft quality and diameter will all affect the mean graft flow (MGF). The “PI” calculates the resistance in the graft and the distal target vascular run-off, respectively. The difference between the peak systolic flow and the peak diastolic flow is subtracted from the median flow to get its value (PI). Indicative values of a MGF of 20 mL/min and a pulsatility index (PI) of 5 were suggested in an expert opinion statement (6). A PI of > 5 and an MGF of 20 ml/min may suggest “technically unacceptable grafts.” Once it was established, TTFM helped to identify a good site for the RCA ligation. By the use of TTFM, excellent MGF was ensured and ischemic events were prevented.

## Conclusion

The intraoperative use of TTFM could be set in guiding the surgeon’s hand making safe, easy and quick the surgical treatment for ACAOS with OPCAB.

## Data availability statement

The raw data supporting the conclusions of this article will be made available by the authors, without undue reservation.

## Ethics statement

Ethical review and approval was not required for the study on human participants in accordance with the local legislation and institutional requirements. The patients/participants provided their written informed consent to participate in this study and for the publication of this case report. Written informed consent was obtained from the individual(s) for the publication of any potentially identifiable images or data included in this article.

## Author contributions

FJ and AL drafted the manuscript and designed the study. FG, ML, CB, and VA were responsible for the collection of data and analysis. FJ, GS, and PM revised the manuscript for significant intellectual content. All authors contributed to editorial changes in the manuscript.

## Abbreviations

ACAOS: anomalous origin of the coronary artery from the opposite sinus of Valsalva
OPCAB: off-pump coronary artery bypass grafting
PI: pulsatility index
R-ACAOS: right ACAOS
RCA: right coronary artery
RIMA: right internal mammary artery
TTE: transthoracic echocardiogram
TTFM: transit time flow measurement.
